# The Semeiology of the Urine; or Treatise on the Alterations Produced in the Urine by Disease; to Which Is Added a Treatise on Bright's Disease at the Different Periods of Life

**Published:** 1842-04

**Authors:** 


### ART. XI.

*Séméiotique des Urines, ou Traité des Altérations de l'Urine dans les Maladies ; suivi d'un Traité de la Maladie de Bright aux divers âges de la vie.* Par ALF. BECQUEREL, M.D. &c. &c.—*Paris*, 1841. 8vo, pp. 576.

*The Semeiology of the Urine; or Treatise on the Alterations produced in the Urine by Disease; to which is added a Treatise on Bright's Disease at the different periods of life.* By ALF. BECQUEREL, M.D. &c. &c.—*Paris*, 1841.

THE manuscript of the present article has been lying on our table for upwards of seven months. The delay arose from our wishing to have an analysis of the first part of the work, especially devoted to the general symptomatology of the urine, incorporated with that of the author's treatise on Bright's disease. Circumstances unfortunately preventing us from being able to give, even now, the notice of that part, we are determined to defer no longer the promulgation of M. Becquerel's views upon a malady which must always be one of much interest to the profession in Great Britain, and which we have reason to believe is at present occupying the attention of not a few active young enquirers. The portion of the volume which we now review is perfectly complete in itself.

M. Becquerel having obtained by his own labour, or by the gift of his friends' portfolios, ninety-four cases of Bright's disease, "the greater part of them curious and interesting, and each complete in itself," found himself tempted strongly to trace the history of the disease in all its relations. The result of this temptation is the treatise before us. The author calls upon his readers to pronounce whether the results he has obtained bear the stamp of novelty, and this with a confidence of tone which, whether it give proof of good taste or not on the part of the appellant, at all events allows those watching over the progress of pathology no excuse for delay in occupying the judgment seat. He conciliates us, however, at the outset of his task by adopting the phrase *Bright's disease* for the malady on the following grounds: because he was unwilling to manufacture a term significative of the morbid change which he himself supposes characteristic of the disease; because that phrase honorably commemorates the discoverer of the malady; because it makes no assumption with regard to the nature of the disease It is natural we should consider these reasons judicious; for the same motives led us to adopt the same conduct in former articles on the subject.

An opening historical sketch of the rise and progress of knowledge on the subjects of dropsy dependent upon renal disease, of albuminous urine, and of Bright's disease itself is sensibly drawn up; and there is probably more soundness in M. Becquerel's rejection of certain vague and random phrases of Hippocrates, as demonstrating any acquaintance on the part of the father of physic with the conditions in question, than in the readiness of MM. Lallemand and Rayer to recognize in those phrases the evidence of such knowledge.

M. Becquerel commences his anatomo-pathological chapter with an enquiry into the external and textural characters of the kidneys differing from the healthy type, yet not necessarily indicative of actual disease in those organs, being either accidental attendants upon such disease, or the result of functional or other changes in distant parts. An enquiry of the kind is manifestly a very important preliminary step in the investigation of the anatomical characters of disease in every structure. It is a point of importance to be able to affirm that undue coloration on the one hand, or an anemic tint on the other, are not the produce of local disease, but of the mode of death, of the constitution of the subject, of the condition of the atmosphere in respect of temperature and humidity, &c. Simple anemic discoloration of these organs has, we doubt not, been mistaken for one of the phases of Bright's disease, and for this reason it may not be without its utility to dwell somewhat upon its anatomical characteristics as described by the author.

The colour of the anemic organ is yellowish, with occasionally a shade of white. The pyramids of Malpighi (tubular cones) often share in this discoloration, but almost always present a deeper tinge, and the most external and widest part of the cones very commonly possesses a red hue, forming a sort of border of a deeper colour than the rest. It is a difficult point to explain the accumulation of blood in this particular situation; it probably depends upon some anatomical arrangement as yet undiscovered. If the state of anemia be complete, the cortical substance is completely discoloured; this is however rarely the case under ordinary circumstances; it is most common to find a few vascular arborizations in the external vascular rete, as also in the longitudinal striæ of the cortical substance. This state of the kidney may be distinguished from the discoloration of Bright's disease by the absence of enlargement, softening, and the peculiar chamois-leather tint observable in the latter case. The absence or presence of the essential characteristics of Bright's disease, characteristics to be presently discussed, also serve to distinguish the two conditions.

M. Becquerel, at a very early stage of his investigations, recognized the general accuracy of the descriptions given by M. Rayer, of the gross changes arising in the renal tissues during the course of Bright's disease, and accordingly devoted his attention to the discovery of the essential element of those changes. This he is persuaded exists in the glands of Malpighi. The changes arising in these minute bodies are referrible to three stages, which comprise, as follows, the six phases admitted by M. Rayer.* The first is a sanguineous congestion and distension of the glands, whence arise the redness and enlargement characteristic of that stage; the fact of this glandular congestion (recognized by M. Rayer himself) may be easily ascertained with the help of a common lens, but that the enlargement is dependent upon the change in these glands appears more distinctly, as shown by M. Becquerel, upon examining a kidney which has been submitted to ebullition or plunged into alcohol or dilute nitric acid. By these processes the glands are *coagulated,* and the important part they play in the disease, as it is alleged, distinctly shown. We have not, we confess, made these experiments, but admitting that the result announced will invariably follow, we cannot see that the fact proves these glandular bodies to be the *exclusive* seat of *injection,* as M. Becquerel would teach us to believe.

The author's second stage corresponds to the second and third of M.

* See the table given of these phases at p. 306 of our Tenth volume.

Rayer. Here the glands of Malpighi are said to be enlarged from infiltration with a yellowish material, which might with some reason be considered the albumino-fibrinous part of the blood, minus the colouring matter. The cortical substance now appears swollen, yellow, shining, and sometimes presents even a gelatiniform aspect. If a portion of it be boiled, the glands infiltrated with the albumino-fibrinous matter coagulate, and as the mode of this coagulation differs from that of the adjacent cellular membrane, they may then be disengaged with facility from their surrounding connexions. At least this is the ordinary result, but it may happen that the glands contract during the progress of ebullition, and hence, instead of appearing enlarged, actually present less than their average dimensions. The albuminous matter contains not an atom of pus; rubbed down carefully in distilled water and examined under the microscope, not a single pus-globule is to be found; and treated with sulphuric æther, it gives no evidence of the presence of fat. When thus diseased the glands are as nearly as possible impermeable by the finest species of injection.

In the stage of granulation described by Dr. Bright, M. Becquerel finds the glands of Malpighi still further diseased. Their yellowish colour is now exchanged for white, and to this alteration he ascribes their being easily visible, even on careless examination; they are also now still further enlarged.

The fifth stage of M. Rayer is, according to the observation of M. Becquerel, generally an attendant upon such cases as have run a very rapid course. He believes that in this form of the disease all the glands are not implicated, and that those which are diseased, present an unusually marked appearance from the non-implication of the surrounding tissue. He entertains not a shadow of doubt that the appearance of semolina granules ("grains de semoule,") under the capsule depends upon albuminous infiltration of enlarged glands. Occasionally there is an appearance of prolongation of the albuminous matter into the uriniferous ducts.

The main characteristics of the sixth degree of M. Rayer are the hardness of the kidney, and the comparative paucity of granulations. The hardness M. Becquerel explains by the increasing consistence of the albuminous exudation. The absence of granulations in numerous spots is ascribable to the following cause: in proportion as the glands present a more advanced amount of disease, the inter-glandular tissue undergoes increasing pressure and ultimately disappears; the granulations then come in contact with, and may become adherent to, each other. Now as their colour is the same in every direction, granulations cease to be perceptible except in some situations where the morbid change in the glands has not reached its maximum; the surface is the situation in which they are commonly wanting, a fact lending support to the theory just tendered, for there the disease commences and most rapidly attains to full development. Two cases are given exemplifying these notions. A woman died at la Charitè with dropsy and albuminous urine; the disease was of long standing. The kidneys were hard, resisting, and tuberous, the cortical substance exhibiting a uniformly scirrhoid aspect, shining, somewhat gelatinous and without any trace of granulations. By ebullition and the action of nitric acid, each separate minute gland was coagulated, and the entire substance shown to consist of an accumulation of these.

Fibrinous infiltration of the glands of Malpighi constitutes then, according to this author, the *fundamental* phenomenon of Bright's disease; but he does not maintain these bodies to be the exclusive seat of anatomical change. To their enlargement is due the general increase in the dimensions of the cortical substance and of the prolongations which it sends between the tubular cones; and if still further enlargement of the glands follow, separation, disaggregation, and even partial disappearance of those cones is the result. Another important effect of the altered size of the glands is atrophy of the cellular tissue and obliteration of the blood-vessels from pressure, this obliteration is rendered manifest by the colour of the part, by the microscope and by the results of injection. From some experiments made by M. Becquerel on the latter point, it appears that the glands are very imperfectly penetrated even by the finest substance, and even the venous and urinary vessels were in part blocked up.

The rudiments of this plausible and, we confess, to us attractive theory of the disease, are to be found in the writings of M. Becquerel's predecessors. Thus Gluge wrote in 1838: "The essence of the renal degeneration discovered in certain cases of dropsy consists in a disturbance of the circulation in the capillary vessels of the cortical substance, and primarily in the corpora Malpighiana. This disturbance, in turn, consists in interruption of the circulation, the blood-globules lose a part of their substance, become agglomerated, and no further motion of the blood is observed in vessels thus affected." To our author, nevertheless, belongs—should ulterior observation prove the soundness of his views—the great merit of distinctly stating the nature and progress of the anatomical changes, tracing their catenation and establishing the relations of the elementary alterations of texture to the grosser physical conditions which have hitherto mainly engaged the attention of observers. The results whereat M. Becquerel has arrived in respect of Bright's disease, correspond remarkably with those which he some time back obtained from his investigations into the nature of cirrhosis of the liver.* This disease he found constituted by enlargement or hypertrophy of the secreting lobules of the organ, through infiltration with albumino-fibrinous plastic matter, which is at first of soft consistence, [first stage of the affection:] this plastic exudation losing a portion of its contained water, contracts upon the tissue it infiltrates, and thus determines atrophy and shrivelling of the liver, [second stage.] The analogy between cirrhosis and Bright's disease, according to the author's notions of these organic maladies, is sufficiently apparent: his theory of the former is fundamentally the same as that taught by Dr. Carswell,† though in the details he differs from that eminent pathologist. The discrepancy probably in some measure depends upon the still unsettled state of opinion among the most distinguished anatomists, as to the ultimate structure of the liver.

M. Becquerel enquires why the condition of the kidneys should be, as it is known to be, extremely different in cases wherein the symptoms have been nearly alike, and appears to find a solution of the difficulty to his satisfaction in the acute or chronic state of the malady, and the variable amount of vital power of resistance possessed by different indi-

* Vide Br. and For. Med. Rev. vol. X. p. 560. Oct. 1840.

† Illustrations of the Elementary Forms of Disease, fascic. *Atrophy*.

viduals. He denies that the disease is, as maintained by M. Rayer, an inflammation, because the anatomical characters of that state, redness and swelling, friability, softening, and formation of pus, have never been observed as its attendants. The existence of congestion by no means involves that of inflammation; in the instance of Bright's disease hyperemia rarely originates in acute change, but commonly proceeds from the habitual stagnation of blood in the abdominal viscera consequent on disease of the heart, pulmonary emphysema, &c.

Such anatomical changes as arise in the kidney, independently of those of the corpora Malpighiana, are esteemed by M. Becquerel mere complications of the essential malady. Thus he regards in this light the induration of the cellulo-vascular tissue which separates the bodies just referred to; an induration evidently contributing to produce those irregularities of shape so commonly observed in kidneys affected with Bright's disease. Here appears also adhesion of the external capsule to the substance of the organ, with the milky-white patches frequently coexisting therewith. The ecchymotic red spots sometimes observed on the yellow ground, are by M. Becquerel presumed to depend not upon morbid vascularity, but simply upon the continued permeability of some few vessels. From 84 dissections recorded by Christison, Rayer, Martin-Solon, Forget, Tissot, &c. and 45 made by himself, he has ascertained the following to be the relative frequency with which the different stages of the malady are found at the period of death.

| | | |
|---|---|---|
| First degree, | (first of M. Rayer,) - - | 10 |
| Second degree, | (second and third of do.) - | 41 |
| Third degree, | (fourth, fifth, and sixth of do.) | 78 |
| | | 129 |

M. Becquerel's account of the secondary lesions of the affection acquires value from its numerical statements of the relative frequency of these. For example: Effusion into the peritoneum was detected in 96 subjects; into the pleura in 65; into the pericardium in 36 of the group of 129 just referred to. For further information of the same kind we refer to the original; the chapter is otherwise unmarked by novelty.

In the examination of the organic lesions, which may be considered to be developed previous to or at the same time as the renal malady, cardiac affections hold a prominent place. This is a just tribute to their frequency; M. Becquerel's figures show, that in about a third of the cases of Bright's disease some form of lesion, more or less characteristic, exists in the heart or valves. Now it is remarkable, as the author observes, that notwithstanding the equally great frequency of tuberculous formation in the victims of the renal affection, cardiac and tuberculous disease so rarely coexist in these subjects, that they almost seem mutually exclusive. We are unable to assist the author or our readers to an explanation of the fact—for such we may pronounce it to be on the faith of the analysis of his cases given by M. Becquerel.

In 51 of the 129 subjects already referred to, tuberculous disease of the lungs was detected; but in 29 only of these did the state of the pulmonary disease appear sufficiently advanced to justify the notion of its having exercised any material influence on the production of the renal disorganization.

M. Becquerel discovered in 15 of the 45 subjects dissected by himself, cirrhosis of the liver—still in its first stage in 9, advanced to its second in 6 of these. In 9 cases the hepatic and renal maladies were complicated with an organic lesion, probably of earlier date, of the lungs or heart. In 3 cases the cirrhosis was very far advanced, had evidently preceded in order of development, and probably acted as the cause of the disease in the kidneys. In the three remaining subjects both organs appeared in equally advanced stages of anatomical change.

In enumerating the changes undergone by the liquids in Bright's disease, the author states, that in addition to the blood found in the urine in certain stages of the disease, this fluid occasionally contains an insoluble organic matter of undetermined nature, exhibiting itself under the microscope in the form of amorphous and irregular fragments of a brownish colour; and further conjectures that this matter may consist of the colouring material of the blood in an altered condition. M. Becquerel agrees with his predecessors in recognizing the diminution of the normal chemical principles of the urine, (he terms urine thus characterized *anemic,* because it is observed in an anemic state of the economy,) as averred by them to exist in the disease; but he differs from them in maintaining that the decrease in amount of urea, does not proportionally exceed that of the other ingredients, and in regarding it as a simple condition of the existence of an *anemic* state of the urine, and not of the presence of Bright's disease. Another species of urine described in the earlier part of the volume, under the title *febrile,* is also observed in Bright's disease, when intercurrent phlegmasiæ arise—a fact of no mean importance, inasmuch as it shows that extensive disease of the kidneys does not prevent the fluid they secrete from undergoing the ordinary changes consequent upon variations in the state of the organism in general. Urine secreted by kidneys affected with the malady under consideration, sometimes become *alkaline;* this arises, as is alleged, from the decomposition of urea, and the development of carbonate of ammonia especially: an explanation already objected to by M. Martin-Solon. (Vid. Brit. and For. Med. Rev., vol. X. p. 311.)

We give in the opposite page, from our author, a very valuable table of the analyses of the urine in six cases of the disease, complicated or uncomplicated.

The altered conditions of the blood next occupy the author's attention. In order to estimate these correctly, the absence or presence of intercurrent inflammations must be taken into account. The results given by the author are derived from the experiments of MM. Andral and Gavarret; we state these in general terms, referring for the exact figures to the original. In the first category of cases, unattended by inflammation in other organs, the quantity of water is slightly increased, that of the globules and of the solid constituents of the serum, especially of the albumen, notably diminished; the proportion of fibrine remains normal. When inflammation in other regions complicates the primary affection, the urine continues as just described, except in regard of its fibrine—this principle obeys the general law of phlegmasiæ established by MM. Andral and Gavarret, that is, it augments in quantity.

| Disease. | Observations. | Qualities of the Urine. | Density. | Quantity secreted. | Quantity of water. | Sum of principal solids. | Urea. | Lithic acid. | Inorganic Salts. | Organic matters. | Albumen. |
|---|---|---|---|---|---|---|---|---|---|---|---|
| Simple. | 1*st. Anal.* A little fever, and march acute. | Colour greenish yellow, somewhat dirty, very acid, a little mucus. | 1016·380 | 816·20 | 787·576 | 28·624 | 9·495 | 0·264 | 5·385 | 3·765 | 9·715 |
| | 2*d. Anal.* Disease become chronic; apyrexia. | Lighter yellowish colour; acid. | 1010·080 | 1030·750 | 1011·610 | 19·140 | 6·520 | 0·616 | 4·277 | 5·097 | 2·630 |
| Simple. | Male on the point of recovery; less albumen vastly than formerly; he got well eventually. | Acid; light colour with greenish shade, very little mucus. | 1007·560 | 1830·400 | 1807·360 | 23·040 | 11·648 | 0·585 | 4·615 | 5·984 | 0·208 |
| With polydipsia. | Male æt. 35, polydipsia œdema of lungs, ascites; improvement with tinct. of cantharid. horse-radish and vapour baths. | Urine light coloured alkaline, whitish deposit, effervescence with acids. | 1008·400 | 2291·200 | 2259·444 | 31·756 | 4·272 | 0·448 | 6·608 | 12·620 | 7·808 |
| With pulm. tubercles. | Male, drinks much, lungs infiltrated, a little ascites. | A little acid, pale, clear, and abundant. | 1005·460 | 3117·500 | 3083·405 | 34·095 | 11·910 | 0·795 | 5·268 | 7·952 | 8·170 |
| In first stage. | No serous effusion of any kind. | Sanguinolent, a little lithic acid, very acid. | 1012·600 | 741·25 | 722·320 | 18·930 | 5·784 | 0·347 | 3·800 | 4·454 | 4·545 |
| | | | 1010·080 | | | 25·931 | 8·322 | 0·509 | 5·159 | 6·645 | |

So much for M. Becquerel's results in respect of the normal principles of the blood; he next enquires whether this fluid undergoes modification by the addition of new elements. He is inclined to deny the existence of urea in the blood of patients suffering under this disease, but admits the point to be one needing further experimental investigation. The motives of his doubt appear in the results of seven most carefully conducted analyses—three of them made by M. Quevenne, two by M. Lecanu, and two by himself; in these instances not the least trace of urea was detected. On the other hand, M. Quevenne discovered some of this principle in the blood of a female affected with cardiac disease and dropsy; the kidneys were not diseased here, and the urine did not contain any sensible quantity of albumen. The reader will do well to compare the statements which we now lay before him with the abstract elsewhere given of the results of MM. Christison, Rayer, Guibourt, and others. (vol. X. p. 309.)

The lactescent condition of the serum noted by Christison and others did not exist in any of the cases observed by the author and MM. Andral and Gavarret; the blood, M. Becquerel alleges, scarcely presents a buff, unless as the attendant upon some inflammatory complication. From all this springs the following summary view of the pathology of the affection: "the cortical substance secretes in Bright's disease a urine, the properties of which are in harmony with the general condition of the individual; but by means of some incomprehensible mechanism, which is the consequence of the morbid state of that substance, it permits a certain quantity of albumen to pass, which of course comes from the blood. The blood becomes impoverished by this continual drainage of albumen; the quantity of globules and of the albumen of the serum diminishes, and hence arises a general condition, which savours of the 'anemic state,' or is itself a particular form of this. Under the influence of this state follow dropsies."

In the next chapter, the etiology of the disease is examined with more philosophy and on sounder principles than in previous works, and it is not a little gratifying to us to find that in contesting the alleged reality of certain of the influences described to be productive of the disease by MM. Rayer, Christison, &c., we were destined to be borne out by the figures of M. Becquerel. Thus, we exposed the utter fallacy of the argument adopted by Dr. Christison to prove the influence of intemperance upon the disease; and we find accordingly, that of sixty-nine subjects examined by M. Becquerel, nine only were given to indulgence in spirituous liquors—and in some of these cases certain internal lesions had preceded, and in all probability played an important part in the development of the malady. Again, in one third only of the 69 cases was the affection traceable to any form of exposure to cold and damp; even this proportion of cases, however, it may be urged, suffices to prove the power of those atmospheric conditions, and we do not deny this; but the smallness of the ratio shows that the extent of that power had been greatly exaggerated. M. Becquerel finds himself authorized in affirming, on the evidence of these cases, that the disease most commonly originates under the influence of preexisting organic lesions, of which the immediate effect is to induce an habitual state of congestion in the kidneys. Our limits will, unfortunately, not permit us to analyse this chapter with the attention it deserves.

We have elsewhere mentioned (vol. X. p. 323,) that Dr. Bright estimated the number of persons affected with the disease, as "at least one in six, if not in four," of those suffering sufficiently to keep their beds; a result obtained by boiling the urine of all the denizens of an hospital indiscriminately,—founded upon the notion that albuminous impregnation is pathognomonic of the disease,—and, finally, quoted with approbation by Dr. Christison. It is needless to point out, a second time, the error of thus attempting to establish the frequency of the disease, more especially as the days of albuminous enthusiasm may now be considered to be on the wane. The reader will receive with satisfaction the statement that M. Becquerel found 1 only in 85 of the patients received into the wards of the Hôpital de la Charité during the year 1839 affected with the disease; he will receive it with satisfaction, we mean, because it is an estimate plainly devoid of exaggeration, and made by an observer who, while he has no theory of his own affected by it directly or indirectly, had made himself master of the diagnosis of the disease. Out of 1448 patients admitted, 31 had pneumonia; 28 pleurisy; 66 continued fever; 38 diseases of the heart; and 17 the affection now under consideration. This calculation does not of course furnish proof that Bright's disease may not be more frequent in this country, but it does to our minds demonstrate that its real prevalence has been enormously overrated.

We pass over the chapters devoted to the symptomatology, the forms and complications of the disease, as comparatively unattractive, and find our attention arrested by the author's judicious statement of the circumstances, independent of the existence of Bright's disease, under which albuminuria may arise. The following extracts contain the most important points in this statement. "All cases in which the existence of albumen in the urine is due to admixture with pus, and the greater number of those in which it depends on the discharge of blood, must be first set aside. However a difficulty occurs here: during the first period of Bright's disease there is often blood in the urine, and it is difficult to determine whether the albumen depends simply on the escape of blood, as in other kinds of hematuria. The point is not always capable of being decided, and in some cases it will be impossible to come to a conclusion, if, as may be the case, this first period be not accompanied with dropsy. Under these circumstances we must wait before venturing upon a decided opinion; for the proportion of the other principles in the urine will throw no light on the matter, inasmuch as all the varieties under which that fluid presents itself, may be met with in the case supposed. No inconsiderable difficulty will be experienced in the following instance also: all dropsies arising as a sequence of scarlatina are not due to disease of the kidneys. Now in dropsy occurring independently of lesion of these organs, it is not very uncommon to observe coexisting hematuria, which determines the escape of albumen with the urine, and the diagnosis becomes so much the more difficult because this fluid has then almost invariably the *anemic* character; and, as I have shown, this is its most common character in Bright's disease also. Once the blood has disappeared, the urine will, it is true, be anemic and cease to contain albumen; but under such circumstances it will be necessary to wait further, in order to be entitled to give a positive opinion."

"Independently of these cases albumen exists in the urine under four principal conditions, and its presence then may render the diagnosis of the existing affection obscure.

"1. Albumen appears in the urine *in the course of* several acute febrile diseases," [the exact terms in which we announced the fact when disputing the correctness of M. Martin-Solon's opinions on the point,] "or of those in which functional disturbance to a certain amount exists. In cases of this kind the quantity of albumen is in general very trifling; it appears very irregularly, disappears to return or to cease completely. Under these circumstances the quantity of albumen is not in general sufficiently great to render the distinction of the case difficult.

"2. Albumen exists in the urine as an attendant upon certain diseases of the heart and some cases of pulmonary emphysema; its presence apparently depends upon mechanical hyperemia of the kidneys. In such cases the quantity of albumen is not greater, or of greater constancy and regularity in its appearance than in the former. If, however, the impregnation become considerable, we are almost warranted in affirming that Bright's disease complicated the cardiac lesion." [Cases on record prove that abundance of albuminous impregnation affords no such warranty.]

"3. Albuminuria exists in certain dropsies depending especially upon a morbid state of the blood, and which, when far advanced, lead to œdema of the kidneys. Such is the state of things sometimes in children, become the subjects of dropsy after scarlatina. Here no affection of the kidneys exists except œdema, and the diagnosis may be rendered obscure by the anemic character almost constantly presented by the urine. The distinction of the cases will be grounded on the fact that in that now referred to, the appearance of albumen does not occur until after dropsy has set in, that the quantity voided is generally much greater than in Bright's disease, and finally that it appears only temporarily, and often irregularly. The diagnosis would be almost impossible, and it would be necessary to procrastinate before pronouncing an opinion, if the quantity of albumen discharged became tolerably abundant, and the observer had had no opportunity of examining the urine from the outset of the disease.

"4. Albumen exists in the urine of some individuals who are perfectly healthy. Upon this it must be observed, that the first symptom of Bright's disease is albuminuria; the morbid changes of the blood and the consequences of these are consecutive phenomena. It may then be the fact, that the presence of albumen announces the development of Bright's disease, although there are no other symptoms of the affection present. The subject so affected appears in the enjoyment of health, but at a later period will appear the characteristic phenomena of Bright's disease. It is therefore necessary to be on the watch in cases where albumen is voided by individuals apparently possessed of sound health. However I observed for six months at the Hôpital de la Charité, a strong, robust, and vigorous infirmary-man, whose urine constantly contained a large proportion of albumen. The other properties of the fluid were normal. He was sent away at the end of six months, but I ascertained he filled the same office at La Pitié a year after. Whether he is destined to have Bright's disease at a future period I am unable to affirm." (p. 557.)

An argument, identically the same in essence as that contained in the last paragraph, was put forth by Dr. Christison, in evasion of the force of M. Solon's discovery regarding the presence of albumen in the urine during febrile diseases. The observations we made on Dr. Christison's attempt are of course applicable to the statement of M. Becquerel.

There is nothing in the author's pages on treatment calling for notice. He is strongly opposed to the use of diuretics, chiefly on theoretical grounds.

A section follows upon Bright's disease as it exists in infants. The anatomical changes are fundamentally the same as at a more advanced period of life, but M. Becquerel has found the granular structure of the cortical substance when in the second and third phases, more distinctly developed in children than in adults. He also describes as follows two forms of change occasionally observed in infants, and which are not precisely similar to those described as affecting subjects who have reached puberty: "1. The size of the kidneys is slightly augmented, the capsule adheres only partially or not at all to the cortical substance. The surface of this substance is somewhat tuberous and irregular, but smooth and of a blueish-white colour; it presents some injected vessels and stellate ecchymoses. Attentive examination shows that this tissue is constituted by an agglomeration of a great number of granulations of blueish-white colour, and sometimes of tolerably large size, many of them equalling that of a pin's head. They are in general heaped up together, pressed against each other, and sometimes united so as to form small white granular patches. On section the cortical substance is seen to be enlarged, and presents in every part, as well as the prolongations into the tubular substance, the change just described; the tubular cones are often notched and partially destroyed. 2. The size of the organs is somewhat increased, the capsule easily removeable exhibits a chamois-leather colour without any injection. It presents an extremely abundant sprinkling of small granulations of similar, but lighter hue, and all of them about as large as a pin's head. They are often provided with a sort of prolongation or small tail."

Such are the principal novelties which this valuable treatise puts forth; we recommend the reader interested in renal pathology, however, not to content himself with the abstract we have just laid before him, but to procure the original work and carefully examine it for himself.

---

